# Evaluating IPMN and pancreatic carcinoma utilizing quantitative histopathology

**DOI:** 10.1002/cam4.923

**Published:** 2016-09-26

**Authors:** Evan S. Glazer, Hao Helen Zhang, Kimberly A. Hill, Charmi Patel, Stephanie T. Kha, Michael L. Yozwiak, Hubert Bartels, Nellie N. Nafissi, Joseph C. Watkins, David S. Alberts, Robert S. Krouse

**Affiliations:** ^1^ University of Tennessee Health Sciences CenterMemphisTennessee; ^2^ The University of ArizonaTucsonArizona; ^3^ University of ColoradoDenverColorado; ^4^ CMC Veterans Affairs Medical CenterPhiladelphiaPennsylvania; ^5^ University of PennsylvaniaPhiladelphiaPennsylvania

**Keywords:** Intraductal papillary mucinous neoplasms, karyometry, nuclear chromatin pattern, pancreatic carcinoma, quantitative histopathology

## Abstract

Intraductal papillary mucinous neoplasms (IPMN) are pancreatic lesions with uncertain biologic behavior. This study sought objective, accurate prediction tools, through the use of quantitative histopathological signatures of nuclear images, for classifying lesions as chronic pancreatitis (CP), IPMN, or pancreatic carcinoma (PC). Forty‐four pancreatic resection patients were retrospectively identified for this study (12 CP; 16 IPMN; 16 PC). Regularized multinomial regression quantitatively classified each specimen as CP, IPMN, or PC in an automated, blinded fashion. Classification certainty was determined by subtracting the smallest classification probability from the largest probability (of the three groups). The certainty function varied from 1.0 (perfectly classified) to 0.0 (random). From each lesion, 180 ± 22 nuclei were imaged. Overall classification accuracy was 89.6% with six unique nuclear features. No CP cases were misclassified, 1/16 IPMN cases were misclassified, and 4/16 PC cases were misclassified. Certainty function was 0.75 ± 0.16 for correctly classified lesions and 0.47 ± 0.10 for incorrectly classified lesions (*P* = 0.0005). Uncertainty was identified in four of the five misclassified lesions. Quantitative histopathology provides a robust, novel method to distinguish among CP, IPMN, and PC with a quantitative measure of uncertainty. This may be useful when there is uncertainty in diagnosis.

## Introduction

Intraductal papillary mucinous neoplasms (IPMN) are a heterogeneous group of pancreatic lesions with uncertain biologic behavior 1. Approximately one out of three lesions will develop into a malignancy, and identifying which cancers have a higher probability to metastasize is of great debate and difficulty 2. As such, lesions of indeterminate or high risk based on imaging are often sent for biopsy or even directly to surgical resection. The overall goal of this research is to develop an objective method to risk stratify IPMN.

It can be difficult to distinguish between malignant IPMN and nonmalignant IPMN on tissue biopsy or even resection unless there is clear invasion demonstrating pancreatic carcinoma (PC) 3, 4. Quantitative histopathology has a well‐described role in classifying premalignant lesions into low or high risk based on numerous nuclear features 5, 6, 7. In our laboratory, we can measure up to 93 unique nuclear features based on standard histopathological slides. We have also demonstrated its utility in distinguishing aggressive malignancies from nonaggressive malignancies 6, 7, 8.

The purpose of this exploratory work is to build novel, objective, and accurate prediction tools to classify pancreatic tissues into three distinct groups using quantitative histopathologic signatures in high‐resolution images of nuclei of histologic sections. We hypothesized that a nuclear signature could properly classify a lesion into chronic pancreatitis (CP), IPMN, or PC based on analysis of H&E slides. CP was chosen as a control arm because CP is a risk factor for PC 9, and there was a relative paucity of benign pancreatic tissue available for analysis. The pathologist's evaluation was utilized as the gold standard comparator in this analysis.

## Materials and Methods

### Patients

**Figure 1:**
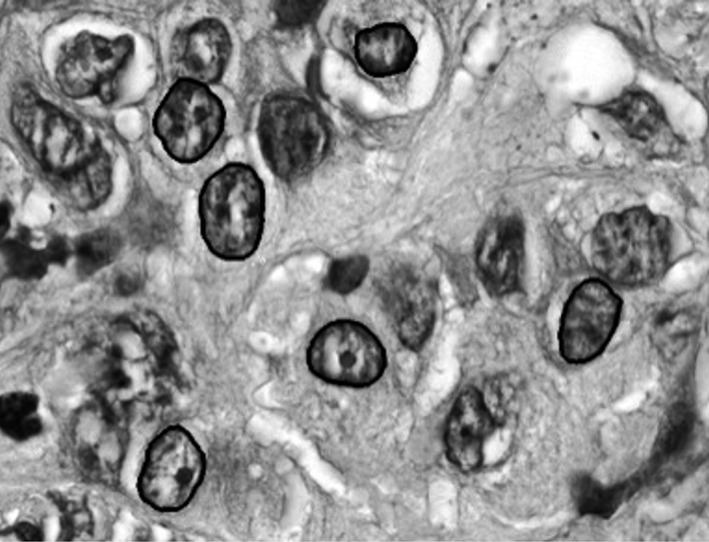
Gray scale image of H&E slide of pancreatic carcinoma demonstrating segmentation of nuclei. A semiautomated imaging algorithm segments the nuclei from surrounding cytoplasm and artifacts. Within a segmented nucleus, each pixel is analyzed and mapped in a grid (*x*‐*y* plane) and analyzed with results seen in Table 2.

Forty‐four patients who underwent pancreatic resections were retrospectively identified. Twelve cases of CP, 16 cases of IPMN, and 16 cases of PC were utilized in this pilot study. Nuclei from each lesion were imaged with high‐resolution microscopy (Fig. 1), and the nuclei were segmented as previously described 6, 10. Clinicodemographic data were obtained from the medical record. Cancer staging was determined by a pathologist according to the American Joint Committee on Cancer Guidelines, 7th edition. The University of Arizona Institutional Review Board approved this project.

### Multiple class lesions analysis

The goal of determining nuclear features that distinguish among CP, IPMN, and PC is to properly characterize each lesion. After determining the statistically significant nuclear features, each lesion was given a probability of being classified as CP, IPMN, or PC based on the average of all nuclei in the lesion. The combined probability score for each of the three classifications must equal 100% (1.0) for each lesion. A lesion was classified based on the highest probability of the three classification groups.

Next, we sought to determine the certainty in which the classification was determined by subtracting the smallest classification probability from the largest probability (of the three classification groups). The certainty function varies from 1.0 to 0.0, with 1.0 being perfectly assigned and 0.0 being assigned by random chance.

### Nuclear features

Statistically significant nuclear features were determined by a fully automated penalized multinomial regression algorithm in order to determine a multiclass classifier and simultaneously identify important nuclear features. The Lasso penalty function 11 was employed for feature selection, and its associated regularization parameter was adaptively chosen by cross‐validation to prevent overfitting 12. In order to test the veracity of the automated algorithm, we randomly sampled 75% of the cases as a training set and utilized the remaining cases as a test set; this was repeated 20 times to estimate overall accuracy.

We conducted the analysis at two levels: one at the tissue level and the other at the nuclear level. At the tissue level, we first pooled the features of nuclei from the same tissue by the sample average and then used the average features for this analysis. At the nuclear level, we did not directly observe the cancer type label for each nuclei since this was a blinded analysis at the nuclear level. Therefore, we first imputed the label for each nuclei using the model obtained from the tissue‐level analysis, and then conducted the nuclear‐level analysis using raw features observed for each nuclei.

Regularized multinomial regression was the method used for the analysis. This is a modern technique for obtaining sparse classification rules in the context of multiple regressions. The standard multinomial regression assumes that the natural log of the odds between each pair of outcomes is a linear function of features.

Since the number of features in our study was large and not all of them were relevant to prediction, we further imposed sparse penalties on the regression coefficients to identify important features. Specifically, in order to optimally identify the most relevant nuclear features, we maximized the penalized log likelihood function subject to the Lasso penalty 11 and the group Lasso penalty 12. The R package “glmnet” was used to analyze the data 13. This method involved a tuning parameter selected adaptively for the data in order to achieve optimal performance. We used cross‐validation to select the tuning parameter based on two types of selection criteria: one based on the deviance measure and the other based on the classification accuracy measure.

### Statistical analysis of group classification

We first conducted analysis based on the whole data and reported the estimated class probabilities, training error, and the selected features. In order to report the future generalized performance, we also randomly split the data set into two parts, the training set and the test set. The training set was used to fit the penalized multinomial regression, and the test set was used to evaluate the classification accuracy of the classifier. We used a 3:1 ratio for the spilt (i.e., three quarters of the data were used for training and one‐quarter for testing). For stabilization, we repeated this split 20 times and reported the average classification accuracy of the test set. The number of the selected important features was also reported.

### Statistical analysis of clinical characteristics

Clinical data were analyzed with group comparisons utilizing Student's *t*‐test or ANOVA, as appropriate. Alpha was assumed to be 0.05. Uncertainties were standard errors of the mean unless otherwise stated.

## Results

### Patients

Forty‐four patients who underwent pancreatic resections were identified: 12 cases of CP, 16 cases of IPMN, and 16 cases of PC. From each lesion, 180 ± 22 nuclei were imaged with high‐resolution microscopy (Fig. 1). Follow‐up was 1.4 ± 0.9 years in the CP group, 2.4 ± 1.5 years in the IPMN group, and 2.2 ± 2.0 years in the PC group. Clinicodemographic data and AJCC Stage data are listed in Table 1. Four patients with PC developed metastatic disease during the follow‐up period; no patients developed recurrence without metastatic disease.

**Table 1:** Clinical and demographic data by diagnosis group

	CP*n* = 12	IPMN*n* = 16	PC*n* = 16
Age			
Mean	47.4	72.0	66.1
Min	34.3	56.5	41.4
Max	61.2	90.6	85.7
Gender			
Male	5	8	13
Female	7	8	3
Race			
Caucasian, *n*	9	14	9
Not Caucasian, *n*	3	2	7
Stage I	n/a	n/a	4
Stage II			10
Stage III			2
Metastatic disease, *n*	0	0	4

CP, chronic pancreatitis; IPMN, intraductal papillary mucinous neoplasms; PC, pancreatic carcinoma.

### Classification algorithm

**Figure 2:**
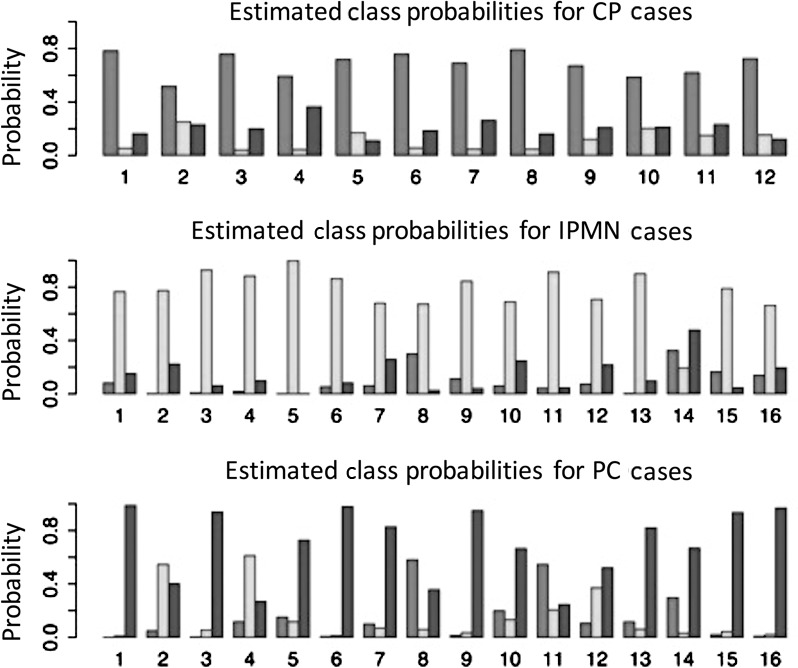
The probability that a lesion was identified as CP (medium gray), IPMN (light gray), or PC (dark gray) is demonstrated for each lesion regardless of its true diagnosis. Proper lesion classification, as defined by the maximum probability of the three options, was achieved in 89.6% of lesions, with one IPMN and four PC misclassified. CP, chronic pancreatitis; IPMN, intraductal papillary mucinous neoplasms, PC, pancreatic carcinoma.

For the whole data analysis, the Lasso method classified CP cases perfectly, misclassified one out of 16 patients with IPMN, and misclassified four out of 16 patients with PC. The overall total classification accuracy was 89.6% with six unique features. The group Lasso achieved the same accuracy with six features (five of the six features being the same). To provide an uncertainty measurement for classification results, we also reported the estimated class probabilities for each sample (Fig. 2). The figure contains three rows—one for each diagnosis (pathology gold standard)—and the height of each bar reflects the probability value of the sample belonging to one class (medium gray for CP, light gray for IPMN, and dark gray for PC).

To assess the future prediction accuracy, we conducted the random split 20 times and reported the average result. With regard to cancer classification accuracy, the method with the Lasso penalty achieved 83.2% classification accuracy on the test set; with the group Lasso penalty, the classification accuracy was 82.7%. With regard to feature selection, the method with either Lasso or group Lasso identified 7.55 features on average. For the nuclei‐level analysis, the classification and feature selection results were similar to the tissue‐level analysis.

### Lesion identification

Six nuclear features were distinguished among the three groups (Table 2). The overall correct classification was 89.6% (39/44, Table 2). All 12 CP lesions were classified as CP lesions, while 15/16 (94%) IPMN and 12/16 (75%) PC were correctly classified.

**Table 2:** Nuclear features that distinguish among chronic pancreatitis (CP), intraductal papillary mucinous neoplasms (IPMN), and pancreatic carcinoma (PC)

Nuclear features	CP*n* = 12Mean (SD)	IPMN*n* = 16Mean (SD)	PC*n* = 16Mean (SD)
Nuclear roundness	1.63 (0.04)	1.79 (0.10)	1.69 (0.04)
Run length matrix	12.80 (1.16)	10.08 (2.44)	10.85 (1.98)
Short run emphasis	0.51 (0.03)	0.47 (0.04)	0.46 (0.03)
Long run emphasis	9.83 (0.91)	11.50 (0.86)	10.58 (0.84)
Run percentage	467.37 (47.33)	511.37 (128.25)	723.86 (165.97)
Total number of lightly stained pixels	230.17 (51.18)	340.87 (171.84)	581.29 (227.73)

**Figure 3:**
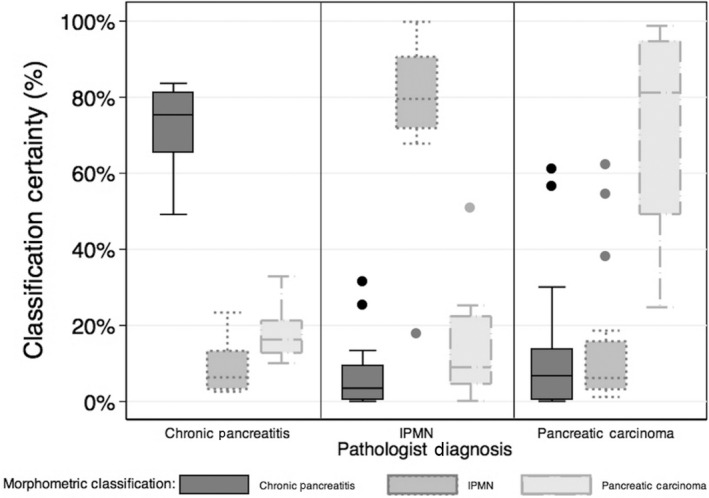
The aggregate data demonstrate that quantitative histopathology classifies lesions into CP, IPMN, and PC lesions. CP, chronic pancreatitis; IPMN, intraductal papillary mucinous neoplasms; PC, pancreatic carcinoma.

**Figure 4:**
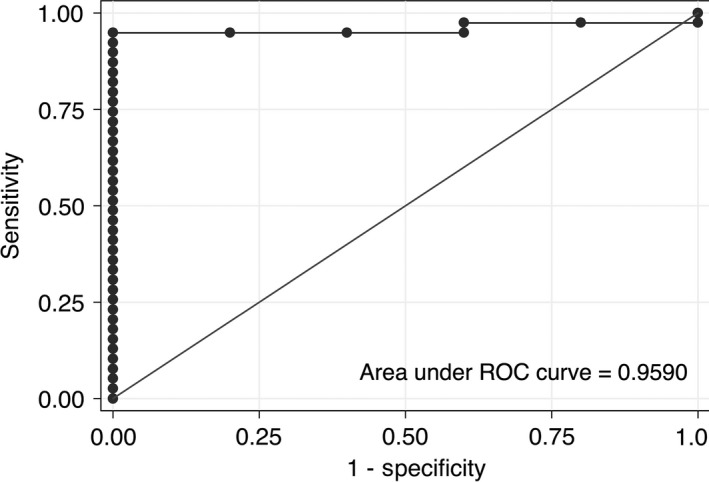
The area under the ROC curve for proper classification based on the maximum probability of chronic pancreatitis, IPMN, or pancreatic carcinoma is 0.96 ± 0.03. The comparison *gold standard* is pathologist diagnosis of those three diagnoses.

The certainty function was 0.75 ± 0.16 for correctly classified lesions (*n* = 39) and 0.47 ± 0.10 for incorrectly classified lesions (*P* = 0.0005). In general, the certainty function was >0.6 (equivalent to 60%) for properly classified lesions in each group (Fig. 3). Of the five patients who were misclassified, one had an IPMN lesion and four had PC. Overall, four of these lesions had diagnostic uncertainty with a certainty function score of <0.55. Three patients with CP (all properly classified) had certainty function scores <0.55. Of the patients with PC, the certainty score was 0.78 ± 0.21 for patients not developing metastatic disease and 0.58 ± 0.05 for those who did eventually develop metastatic disease (*P* = 0.086). Finally, the area under the receiver operating characteristic curve testing the classification algorithm (highest probability) compared to gold standard pathologist analysis was 0.96 ± 0.03 (Fig. 4).

## Discussion

Pancreatic carcinoma remains one of the deadliest cancers in the USA with curative resection resulting in a <25% 5‐year survival and unresectable patients' 5‐year survival rates <5%. As such, the clinical algorithm for management of premalignant lesions is aggressive, and intensive therapy often results in major morbidity and mortality 14. Since the morbidity and mortality of the pancreaticoduodenectomy is so high, it is critically important to optimize patient selection. Identifying patients with IPMN who are at the greatest potential for benefit remains a clinical challenge.

The role of quantitative histopathology is yet to be fully utilized, but we have demonstrated that it may be a robust and efficient means to distinguish between IPMN and PC, even in the context of CP. The results herein support the use of quantitative histopathology to help guide surveillance practices, such as more frequent imaging in high‐risk patients. Likewise, if quantitative histopathology further demonstrates diagnostic utility in fine‐needle aspirate samples, then the next logical step would be to develop and/or integrate this technique into a diagnostic tool.

Linder et al., 15 demonstrated the value of a limited number of nuclear morphometric measurements in predicting long‐term survival of patients with unresectable pancreatic carcinoma. We demonstrated that quantitative histopathology (and the associated signature of 6–8 nuclear features) is approximately 89% accurate in classifying lesions and 100% accurate in identifying CP. With a c‐statistic of 0.96, this is a very robust and sensitive test, especially given the exploratory nature of this study. The value of this technique is less in classifying resected specimens, but more so in classifying and risk‐stratifying biopsy specimens prior to pancreatic resection. The pathologist's evaluation was utilized as the gold standard comparator in this analysis such that in the future, quantitative histopathology may assist pathologists in identifying lesions or biopsy samples that are difficult to characterize.

Importantly, quantitative histopathology is quite generalizable. It can be performed on routine H&E‐stained samples using standard high‐resolution microscopy. Furthermore, the actual image analysis was performed on a standard computer. In the future, this could be performed at a central location with HIPAA compliant software and image transfer, if needed. Finally, if needed, slide samples or tissue blocks could also be shipped to a central laboratory for analysis as is commonly performed for other biologic tests.

It can be difficult to distinguish IPMN from PC on a biopsy specimen when there is not clear invasion. This is especially the case when there is significant inflammation such as when CP or fibrosis is present 16, 17. The purpose of this work was to demonstrate the proof of principle in using histopathological and statistical technique to distinguish among CP, IPMN, and PC. Quantitative histopathology may assist pathologists in risk‐stratifying patients with ambiguous pathology.

Limitations of quantitative histopathology exist in two distinct types. First, sampling error will never be resolved. Second, while quantitative histopathology identified CP with 100% accuracy, of the five misclassified lesions, four were PC. This is somewhat concerning because missing a benign lesion results in overtreatment, but missing PC would result in undertreatment and potentially missing a resectable cancer. As such, the utility of quantitative histopathology may be in confirming low risk in patients with low pretest probability for PC. The next logical extension of this research in establishing the utility of quantitative histopathology on resected specimens would be to evaluate fine‐needle aspirates acquired prior to resection. While conventional wisdom is to assume the worst with pancreatic lesions, there is clearly a role for an objective, novel method to risk stratify patients. These limitations are tempered by the fact that long‐term survival remains minimal in patients with PC, even with purported curative resection.

A final limitation is the difficulty in the generalizability of this technique and the technical aspects of analysis. It requires a team approach among the oncologist, pathologist, image acquisition team, and data analysis. While this technique could be performed in a “tele‐pathology” manner, there are certainly difficulties in establishing this analysis technique in other institutions. However, as is demonstrated here, the team approach helps resolve these limitations.

The use of six variables in the classification function, when applied to the very small‐sized samples for both training and test sets, may constitute an overfitting with too low a sample size‐to‐dimensionality ratio. Since overfitting may result in overly optimistic correct classification rates, an independent analysis was carried out for quality control purposes. This analysis compared results from an overfitted, case‐based approach with those from a nuclear population‐based approach with adequate sample size‐to‐dimensionality ratio. It established that the IPMN and the PC data sets were so distinctly different that a high correct classification rate was attained by either approach. Therefore, for these data sets, the overfitting demonstrated no distinct effect.

Likewise, the certainty function quantitates the uncertainty that exists in a given classification. The certainty function identified uncertainty (score <0.55) in four of the five misclassified lesions. While the true utility of this algorithm needs to be demonstrated in a second, and ideally prospective cohort, these results suggest that even on biopsy, quantitative histopathology may yield insightful and useful data for properly risk stratifying patients with pancreatic neoplasm.

Quantitative histopathology classifies pancreatic lesions into CP, IPMN, and PC with 89.6% accuracy using a fully automated algorithm to determine statistically significant and unique nuclear features. Since the incorrectly classified lesions had a larger proportion of mixed nuclei, diagnostic uncertainty may be determined in a quantitative manner allowing for a confidence probability estimation of whether a given lesion should be classified as CP, IPMN, or PC. Further studies will validate these results in a resected cohort as well as a cohort based on biopsied specimens alone.

## Conclusions

Quantitative histopathology provides a robust, novel method to distinguish among patients with CP, IPMN, and PC. This may be useful when there is diagnostic uncertainty. In addition, future work will evaluate the utility of quantitative histopathology in diagnosing pancreatic masses using fine‐needle aspirate biopsies. Finally, the certainty function score yields a quantitative measure of how much uncertainty exists in a given classification.

## Conflict of Interest

DSA and PHB have patents relating to karyometry in measuring lesion progression toward malignancy. The other authors declare that they have no conflicts of interest.

## References

[1] Correa‐Gallego, C. , C. R. Ferrone , S. P. Thayer , J. A. Wargo , A. L. Warshaw , and C. Fernandez‐Del Castillo . 2010 Incidental pancreatic cysts: do we really know what we are watching? Pancreatology 10:144–150. 20484954 10.1159/000243733 PMC3214832

[2] Werner, J. , S. Fritz , and M. W. Buchler . 2012 Intraductal papillary mucinous neoplasms of the pancreas‐a surgical disease. Nat. Rev. Gastroenterol. Hepatol. 9:253–259. 22392299 10.1038/nrgastro.2012.31

[3] Remotti, H. E. , M. Winner , and M. W. Saif . 2012 Intraductal papillary mucinous neoplasms of the pancreas: clinical surveillance and malignant progression, multifocality and implications of a field‐defect. JOP 13:135–138. 22406584

[4] Augustin, T. , and T. J. Vandermeer . 2010 Intraductal papillary mucinous neoplasm: a clinicopathologic review. Surg. Clin. North Am. 90:377–398. 20362793 10.1016/j.suc.2009.12.008

[5] Bersch, V. P. , V. D. da Silva , A. B. Osvaldt , M. S. da Costa , L. Rohde , and D. Mossmann . 2003 Digital karyometry in pancreatic adenocarcinoma. Anal. Quant. Cytol. Histol. 25:108–114. 12746980

[6] Glazer, E. S. , P. H. Bartels , A. R. Prasad , M. L. Yozwiak , H. G. Bartels , J. G. Einspahr , et al. 2011 Nuclear morphometry identifies a distinct aggressive cellular phenotype in cutaneous squamous cell carcinoma. Cancer Prev. Res. (Phila.) 4:1770–1777. 21636541 10.1158/1940-6207.CAPR-10-0404 PMC3181389

[7] Krouse, R. S. , D. S. Alberts , A. R. Prasad , M. Yozwiak , H. G. Bartels , Y. Liu , et al. 2009 Progression of skin lesions from normal skin to squamous cell carcinoma. Anal. Quant. Cytol. Histol. 31:17–25. 19320189 PMC4061044

[8] Bartels, P. H. , and H. G. Bartels . 2009 Discriminant analysis. Anal. Quant. Cytol. Histol. 31:247–254. 20701090

[9] Ruckert, F. , T. Brussig , M. Kuhn , S. Kersting , A. Bunk , M. Hunger , et al. 2013 Malignancy in chronic pancreatitis: analysis of diagnostic procedures and proposal of a clinical algorithm. Pancreatology 13:243–249. 23719595 10.1016/j.pan.2013.03.014

[10] Bartels, P. H. , R. Montironi , M. Scarpelli , H. G. Bartels , and D. S. Alberts . 2009 Knowledge discovery processing and data mining in karyometry. Anal. Quant. Cytol. Histol. 31:125–136. 19634783 PMC4029779

[11] Tibshirani, R. 1996 Regression shrinkage and selection via the Lasso. J. Roy. Stat. Soc. Ser. B (Methodological) 58:267–288.

[12] Yuan, M. , and Y. Lin . 2006 Model selection and estimation in regression with grouped variables. J. Roy. Stat. Soc. Ser. B (Methodological) 68:49–67.

[13] Friedman, J. , T. Hastie , and R. Tibshirani . 2010 Regularization paths for generalized linear models via coordinate descent. J. Stat. Softw. 33:1–22. 20808728 PMC2929880

[14] Glazer, E. S. , A. Amini , T. Jie , R. W. Gruessner , R. S. Krouse , and E. S. Ong . 2013 Recognition of complications after pancreaticoduodenectomy for cancer determines inpatient mortality. JOP 14:626–631. 24216548 10.6092/1590-8577/1883

[15] Linder, S. , J. Lindholm , U. Falkmer , M. Blåsjö , P. Sundelin , and A. von Rosen . 1995 Combined use of nuclear morphometry and DNA ploidy as prognostic indicators in nonresectable adenocarcinoma of the pancreas. Int. J. Pancreatol. 18:241–248. 8708396 10.1007/BF02784948

[16] Chandan, V. S. , C. Iacobuzio‐Donahue , and S. C. Abraham . 2008 Patchy distribution of pathologic abnormalities in autoimmune pancreatitis: implications for preoperative diagnosis. Am. J. Surg. Pathol. 32:1762–1769. 18779731 10.1097/PAS.0b013e318181f9ca

[17] Malde, D. J. , M. Oliveira‐Cunha , and A. M. Smith . 2011 Pancreatic carcinoma masquerading as groove pancreatitis: case report and review of literature. JOP 12:598–602. 22072250

